# Four suggestions for addressing public concern regarding synthetic biology

**DOI:** 10.1186/1754-1611-4-7

**Published:** 2010-06-09

**Authors:** Alex David Hatch

**Affiliations:** 1 Department of Biological Engineering, Utah State University, 4105 Old Main Hill, Engr. Bldg. Rm. 402 Logan, UT 84322-4105, USA

## Abstract

The following essay was written by Mr. Alex Hatch, a junior undergraduate student majoring in Biological Engineering at Utah State University. Mr. Hatch submitted a 1000-1200 word essay to the 5^th ^Annual Bioethics Contest sponsored by the Institute of Biological Engineering (IBE). A group of professionals in Biological Engineering assessed and ranked the essays in a blinded process. Five semi-finalists were invited to present their essays at a session at the annual meeting of IBE in Cambridge, MA on March 6, 2010. Five judges scored all the presentations and selected Mr. Hatch's contribution as the overall winner (first place).

## Essay

The very characteristics of synthetic biology that make it such a promising field are those that introduce concern. Concerns in the field come from a public sphere and from the scientific community itself [1,2]. As concerns arise, the scientific community has the responsibility to respond in a way that benefits the public first. Addressing all of the concerns facing synthetic biology is beyond the scope of this essay. This essay will focus, rather, on a discussion of the challenges associated with synthetic bioterrorism and a proposal of four steps that can be taken in the short term that would foster safety and trust between the community of synthetic biologists (community) and the public whom they serve.

## Synthetic Biology, Bioweapons, and Bioterrorism

Synthetic biology is "an approach to engineering biology [3]." It builds on the foundational principles of recombinant DNA technologies and allows more efficient modification or enhancement of cellular function [4]. Specific applications that simplify and increase efficiency of the modification of cellular function include but are not limited to:

• Creation of databases of units of DNA that "encode basic biological functions" which are "freely available to the public" [5]

• Development of interchangeable biological parts that are easily combined

• Development and improvements in automated DNA synthesis and whole genome synthesis [6,7]

• Relative ease of ordering DNA parts or synthesized DNA [7]

• Potential to own oligonucleotide synthesizers that would enable independent automated DNA synthesis [8]

As the technology develops, participating in the science will become increasingly efficient and available to a wide range of people [8]. Reports have been released detailing how scientists synthesized pathogenic viruses including polio virus and the 1918 influenza virus [6,9-11]. These examples show that synthetic biology is very powerful and could potentially be manipulated in a malevolent manner.

The Biological and Toxin Weapons Convention prohibits participant nations from using, developing, or stockpiling biological weapons [12]. There are weaknesses in this governing policy, but to this point in time it has prevented international biological warfare. One expert points out, however, that 9/11 demonstrates "that humans [are] capable of unimaginable evil [13]." Moreover, and in contrast to research of the past, the foundational development of synthetic bioweapons could be performed with "little equipment and infrastructure [14]." The intention to use bioweapons and relative ease of implementation could be compatible with terrorist agendas.

## Four Steps to Help Ensure Public Safety

Following are four steps that help address synthetic bioterrorism specifically, but also address concerns regarding synthetic biology. The four steps are:

1.) Community initiative to regulate the receipt of DNA constructs

2.) International regulation

3.) Increased investment in research of risk assessment and public perception

4.) Education

1.) In conjunction with the synthetic biology 2.0 and 3.0 conferences (2006, 2007), suggestions were made to establish best practices for gene synthesis companies [8,15]. These reports indicated that not all gene synthesis companies performed routine screenings for potentially pathogenic DNA orders. Conference participants proposed that the community refuse to do business with those companies that fail to implement routine safety and security practices. A licensing/registration of individuals/equipment needed to perform automated DNA synthesis could take place. The community would then be allowed to determine who was licensed and ensure that known pathogenic constructs were controlled. Required screening and licensing of individuals and automated DNA synthesis equipment would greatly reduce the ability of terrorist groups to obtain harmful constructs and to carry out potentially harmful research. This regulation addresses one of the major public concerns of synthetic biology with little change required in current practices [16].

2.) In dealing with bioterrorism and synthetic biology in general, regulations must exist at an international scale. A failure to have consensus on an international level will lead to difficulties in enforcement. The merging of cultures and backgrounds to create an international policy that meets the demands of contributing parties will be difficult. In the matter of regulating bioterrorism, however, unity must be reached. The attempt for individual nations to regulate will fail because the research can move to a location that has no formal policy against research in question [13]. In an effort to create international policy, the wishes of all participants will never be met, so there must be a willingness to recognize on all sides, fundamental practices that pose a risk to the safety and peace of society, and to act only on those most fundamental principles. International policy accepted in the community of synthetic biology must exist.

3.) While addressing the National Academy of Sciences, David Rejeski stated that approximately 30 million dollars in U.S. federal funding went toward synthetic biology each year [17]. Of the 30 million dollars, he claimed that none was specifically devoted to public engagement or risk assessment. In studies he has overseen, the repeated wish of the public is risk assessment and regulation. Some indicate that at the present, the regulation of recombinant DNA technology also effectively regulates the current standing of synthetic biology [1]. As the science progresses and becomes increasingly novel, increased risk assessment will be vital to safety, funding, and progress. Furthermore, public engagement will be necessary to determine which directions research should and should not take. Increased investment devoted to the study of risk assessment and public perception must take place--especially because the technology is dynamic and progressing--in order to reach the potential that synthetic biology possesses.

4.) While bioterrorism is a valid concern, a major attack using a synthetically derived bioweapon is not realistic at this point in time [18]. The public is introduced to synthetic biology in the media with bioterrorism often mentioned [16]. As Hart Researchers learned in a 2009 survey, about 20% of the public in the U.S. has heard "a lot" or "some" about synthetic biology [19]. Studies show that once an assessment has been made about a risk, the initial assessment rarely changes, but rather becomes stronger [20]. So, as the vast majority of the population is yet to learn about synthetic biology, it is vital that efforts be made to disseminate accurate knowledge to the public through appropriate avenues. Opportunities to discuss possible avenues for education could be discussed at conferences dedicated to synthetic biology, especially the iGEM conferences. Responsibility is taught at such meetings and accepted within the community of synthetic biology. As experts in the field, the teaching and application of safe practices will lead to public trust.

## Conclusion

As synthetic biology evolves, public perception will greatly influence the destination of the science. A major concern of the United States public is bioterrorism introduced with increasingly efficient biological engineering technology. To address this concern four possible steps were suggested. These steps can be used as initial steps to hear and integrate public concerns.

## References

[B1] Rodemeyer M New Life Old Bottles: Regulating First-Generation Products of Synthetic Biology 2009 http://www.synbioproject.org/library/publications/archive/synbio2/31 March, 2010

[B2] Vergano D 'Synthetic Biology' holds promise, but doubts simmer USA Today 2009 http://usatoday.com/news/health/2009-08-30-synthetic-biology_N.htm 31 March, 2010

[B3] Endy D Defining Synthetic Biology 2008 http://2009.igem.org/Instructional_Videos 31 March, 2010

[B4] Rejeski D Foreword-New Life Old Bottles: Regulating First-Generation Products of Synthetic Biology 2009 http://www.synbioproject.org/library/publications/archive/synbio2/31 March, 2010

[B5] BioBricks Foundation http://bbf.openwetware.org 31 March, 2010

[B6] Cello J Paul AV Wimmer E Chemical synthesis of poliovirus cDNA: Generation of infectious virus in the absence of natural template Science 2002 297 1016 1018 10.1126/science.1072266 12114528

[B7] Parens E Johnston J Moses J Ethical Issues in Synthetic Biology: An overview of the debates 2009 http://www.synbioproject.org/library/publications/archive/synbio3/31 March, 2010

[B8] Garfinkel M Endy D Epstein GL Friedman RM Synthetic Genomics - Options for Governance 2007 http://www.synbioproject.org/topics/synbio101/bibliography/governance/31 March, 2010 10.1089/bsp.2007.0923 18081496

[B9] Kurzweil R Joy B Recipe for destruction New York Times A23 (2005, October 17)

[B10] Tumpey TM Basler CF Aguilar PV Zeng H Solorzano A Swayne DE Cox NJ Katz JM Taubenberger JK Palese P Garcia-Sastre A Characterization of the reconstructed 1918 Spanish influenza pandemic virus Science 2005 310 77 80 10.1126/science.1119392 16210530

[B11] Wimmer E The test-tube synthesis of a chemical called poliovirus EMBO Reports 2006 7 S3 S9 10.1038/sj.embor.7400728 16819446 PMC1490301

[B12] Atlas RM Responsible conduct by life scientists in an age of terrorism Science and Engineering Ethics 2009 15 3 263 269 10.1007/s11948-009-9124-7 19421897

[B13] Marchant GE Pope LL The problems with forbidding science Science and Engineering Ethics 2009 15 3 375 394 10.1007/s11948-009-9130-9 19350416 PMC7088679

[B14] Atlas RM Ensuring biosecurity and biosafety through biopolicy mechanisms: Addressing threats of bioterrorism and biowarfare Asian Biotechnol Development Rev 2005 8 121 137

[B15] Maurer SM Lucas KV Terrell S From Understanding to Action: Community-BasedOptions for Improving Safety and Security in Synthetic Biology 2006 http://gspp.berkeley.edu/iths/UC%20White%20Paper.pdf 31 March, 2010

[B16] Pauwels E Ifrim I Trends in American and European Press Coverage of Synthetic Biology 2008 http://www.synbioproject.org/library/publications/archive/why_scientists_should_care/31 March, 2010

[B17] Rejeski D Public Perceptions on the Technological Frontier 2009 http://www.synbioproject.org/process/assets/files/6372/_draft/rejeski.ppt#268,1 Given at National Academy of Sciences July 10, 2009. 31 March, 2010

[B18] Zilinskas R Technical Barriers to Successful Biological Attacks with Synthetic Organisms, Security and Regulation of Experiments of Concern 2006 http://www.synbioproject.org/topics/synbio101/bibliography/governance/

[B19] Hart Research Nanotechnology, Synthetic Biology, & Public Opinion: What does the public think? 2009 http://www.synbioproject.org/events/archive/6380/

[B20] Kahan DM Braman D Braman M Gregory N Risk and Culture: Is Synthetic Biology Different? Harvard Law School Program on Risk Regulation Research Paper No. 09-2; Yale Law School, Public Law Working Paper No. 190 2009 http://ssrn.com/abstract=1347165 31 March 2010

